# Identification of Novel Quantitative Trait Nucleotides and Candidate Genes for Bacterial Wilt Resistance in Tobacco (*Nicotiana tabacum* L.) Using Genotyping-by-Sequencing and Multi-Locus Genome-Wide Association Studies

**DOI:** 10.3389/fpls.2021.744175

**Published:** 2021-10-21

**Authors:** Ruiqiang Lai, Muhammad Ikram, Ronghua Li, Yanshi Xia, Qinghua Yuan, Weicai Zhao, Zhenchen Zhang, Kadambot H. M. Siddique, Peiguo Guo

**Affiliations:** ^1^International Crop Research Center for Stress Resistance, School of Life Sciences, Guangzhou University, Guangzhou, China; ^2^Crop Research Institute, Guangdong Academy of Agricultural Sciences, Guangzhou, China; ^3^Nanxiong Research Institute of Guangdong Tobacco Co., Ltd., Nanxiong, China; ^4^The UWA Institute of Agriculture, UWA School of Agriculture and Environment, The University of Western Australia, Perth, WA, Australia

**Keywords:** tobacco germplasm, bacterial wilt resistance, SNP, genome-wide association analysis, quantitative trait nucleotide, superior alleles

## Abstract

Tobacco bacterial wilt (TBW) is a devastating soil-borne disease threatening the yield and quality of tobacco. However, its genetic foundations are not fully understood. In this study, we identified 126,602 high-quality single-nucleotide polymorphisms (SNPs) in 94 tobacco accessions using genotyping-by-sequencing (GBS) and a 94.56 KB linkage disequilibrium (LD) decay rate for candidate gene selection. The population structure analysis revealed two subpopulations with 37 and 57 tobacco accessions. Four multi-locus genome-wide association study (ML-GWAS) approaches identified 142 quantitative trait nucleotides (QTNs) in E1–E4 and the best linear unbiased prediction (BLUP), explaining 0.49–22.52% phenotypic variance. Of these, 38 novel stable QTNs were identified across at least two environments/methods, and their alleles showed significant TBW-DI differences. The number of superior alleles associated with TBW resistance for each accession ranged from 4 to 24; eight accessions had more than 18 superior alleles. Based on TBW-resistant alleles, the five best cross combinations were predicted, including MC133 × Ruyuan No. 1 and CO258 × ROX28. We identified 52 candidate genes around 38 QTNs related to TBW resistance based on homologous functional annotation and KEGG enrichment analysis, e.g., *CYCD3;2*, *BSK1*, *Nitab4.5_0000641g0050*, *Nitab4.5_0000929g0030*. To the best of our knowledge, this is the first comprehensive study to identify QTNs, superior alleles, and their candidate genes for breeding TBW-resistant tobacco varieties. The results provide further insight into the genetic architecture, marker-assisted selection, and functional genomics of TBW resistance, improving future breeding efforts to increase crop productivity.

## Introduction

Tobacco (*Nicotiana tabacum* L.; 2n = 48) is an important cash crop in many countries, including China, and a valuable model system in genetic engineering and molecular biology. Tobacco bacterial wilt (TBW) caused by *Ralstonia solanacearum* is a destructive soil-borne disease in many regions worldwide (Nishi et al., 2003; Lan et al., 2014; Drake-Stowe et al., 2017). Infected tobacco plants typically exhibit symptoms such as leaf wilt, root and stem necrosis, and growth retardation, followed by plant death, which reduce yield and quality (Qian et al., 2013). TBW is prevalent in tobacco-growing countries with moist tropical or warm-temperate climates (Denny, 2006). In China, the disease occurrence has been steadily rising, reaching 15–30% in some areas (Jiang et al., 2017), posing a serious threat to tobacco production in the four main tobacco-growing regions, including 14 provinces (Li et al., 2016). Several methods, including crop rotation and soil fumigation, can reduce some economic losses from the disease; however, none provide sufficient protection (Nishi et al., 2003; Lan et al., 2014).

Tobacco bacterial wilt resistance is a quantitative trait controlled by multiple genes and/or quantitative trait loci/nucleotides (QTLs/QTNs) (Smith and Clayton, 1948; Nishi et al., 2003; Gao et al., 2010; Ni et al., 2011). It is challenging to improve TBW resistance using traditional breeding methods (Yang et al., 2012). Marker-assisted selection (MAS) is an alternative tool for combining different resistance genes/alleles into a single plant, which has been used to improve different traits in crop breeding programs (Kuchel et al., 2005; Ribaut et al., 2010; Nakaya and Isobe, 2012). It is important to identify significant QTLs/QTNs to develop superior TBW-resistant tobacco cultivars. To date, only four QTL mapping studies for TBW resistance have been conducted in bi-parental and different genetic populations, using simple sequence repeat (SSR) and amplified fragment length polymorphism (AFLP) markers (Nishi et al., 2003; Qian et al., 2013; Lan et al., 2014; Drake-Stowe et al., 2017). Nishi et al. (2003) identified one QTL with 43.8% phenotypic variance using 117 AFLP markers in 125 doubled haploid populations. Similarly, Qian et al. (2013); Lan et al. (2014), and Drake-Stowe et al. (2017) identified four, eight, and two QTLs for TBW resistance, respectively. Thus, only 15 QTLs underlying TBW resistance have been identified, which is relatively small compared to other members of the Solanaceae family (Sharma et al., 2021). Unfortunately, the identified QTLs have large genomic regions unsuitable for detecting candidate genes, and markers linked to these QTLs have limited application in tobacco breeding programs (Li et al., 2017).

With the development of next-generation sequencing (NGS) technology, genotyping-by-sequencing (GBS) has been used widely as a high-throughput and low-cost genotyping platform for discovering genome-wide single-nucleotide polymorphisms (SNPs) in many crops, including tobacco (Elshire et al., 2011; Lee et al., 2017; Sakiroglu and Brummer, 2017). Genome-wide association studies (GWAS) can use these millions of SNPs as molecular markers to screen many accessions simultaneously without needing to construct segregating populations in advance (Buckler and Thornsberry, 2002; Flint-Garcia et al., 2003; Yu et al., 2006; Wang et al., 2016; Zhang et al., 2019). For instance, Thapa et al. (2021) identified 23 and 38 QTNs associated with shoot and blossom blight resistance, respectively, using GBS markers in 273 apple accessions, while Jing et al. (2021) identified 18 QTNs related to Sclerotinia stem rot resistance in soybean. Thus, GWAS is an efficient tool for QTN identification in natural populations with high-quality SNPs to overcome the shortcomings of bi-parental QTL mapping (Zhang et al., 2019) and has great potential for discovering interrelationships among complex traits conditioned by multiple genes/alleles (Buckler and Thornsberry, 2002; Yu et al., 2006; Hyun et al., 2021; Thapa et al., 2021). However, no GWAS studies have been undertaken to detect QTNs associated with TBW resistance in tobacco. Identifying QTNs/alleles/genes related to TBW resistance is an important step for improving tobacco production.

This study assembled a panel of 94 tobacco accessions from seven countries and used GBS sequencing to identify high-density SNPs. The study aimed to: (1) analyze the SNP distribution, linkage disequilibrium (LD), and population structure using GBS data; (2) detect QTNs related to TBW resistance using GWAS; (3) identify TBW-resistant superior alleles of stable QTNs for MAS and the best parental cross combination based on superior alleles; (4) predict potential candidate genes for TBW resistance in the region of stable QTNs. The results of this study will provide information for uncovering the genetic basis of TBW resistance and facilitating MAS in tobacco breeding.

## Materials and Methods

### Plant Material and Phenotyping

Ninety-four tobacco accessions were obtained from the Nanxiong Scientific Research Institute of Guangdong Tobacco Company, China. These accessions came from the United States, Japan, Canada, Somalia, Australia, Zimbabwe, and China, including 90 flue-cured, two sun-cured, and two burley tobacco accessions (Supplementary Table 1).

**FIGURE 1:**
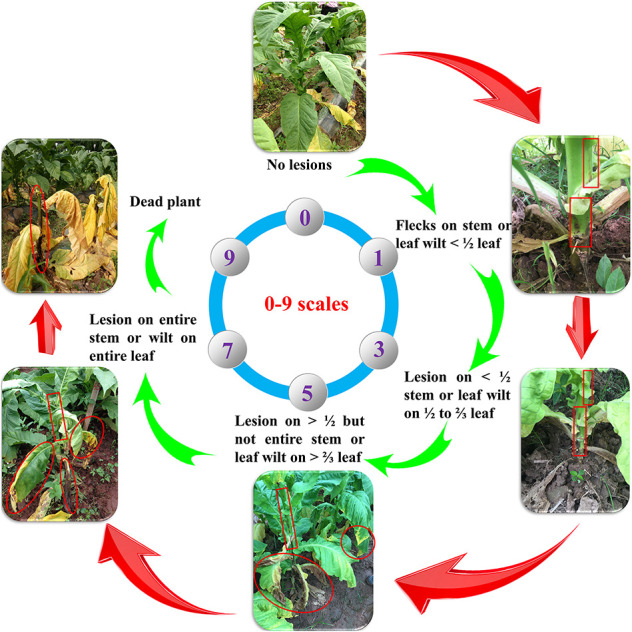
Tobacco bacterial wilt disease rating (0–9 scale).

The 94 accessions were planted at the Hukou experimental station in Nanxiong city in 2013, 2014, and 2015 (denoted E1, E2, and E4) and Xikou experimental station in Nanxiong city in 2014 (denoted E3) in a randomized complete block design with two replicates at each location. Each plot had 20 plants spaced 0.5 m within rows and 1.2 m between rows, with local management practices applied. We used the biochemical type III bacterial wilt pathogen race-1 strain of *R. solanacearum*. The inoculum was applied in early May (May 8, 2013, May 4, 2014, and May 5, 2015) using the stem puncture inoculation method. Disease ratings for each accession occurred on May 30, 2013, May 29, 2014, and May 29, 2015 using the 0–9 scale described in “China National Tobacco Pests Classification and Survey Methods (GB/T23222-2008)”: 0 = no lesions; 1 = flecks on stem or leaf wilt <1/2 leaf; 3 = lesion on <1/2 stem or leaf wilt on 1/2 to 2/3 leaf; 5 = lesion on >1/2 but not entire stem or leaf wilt on >2/3 leaf; 7 = lesion on entire stem or wilt on entire leaf, and 9 = dead plant (Figure 1). The disease index for TBW (TBW-DI) was calculated according to Lan et al. (2014):


TBW-DI=(accession⁢mean⁢rating/9)×100

The disease resistance of the 94 accessions was classified using a 0–100 scale following the standard method of “China National Tobacco Varieties Resistance Identification (YC/T 41-1996)”: 0 < TBW-DI ≤ 25 as highly resistant, 25 < TBW-DI ≤ 50 as moderately resistant, 50 < TBW-DI ≤ 75 as moderately susceptible, and 75 < TBW-DI ≤ 100 as highly susceptible.

### Statistical Analysis of Phenotypic Data

Mean, range, standard deviation (SD), coefficient of variation (CV%), skewness, kurtosis, and analysis of variance (ANOVA) were calculated for TBW-DI of the 94 tobacco accessions in each environment using R4.0.3^1^ software. A best linear unbiased prediction (BLUP) value of TBW-DI for each tobacco accession was calculated using the *lme4* (Bates et al., 2014) statistical package of R. The mixed linear model (MLM) was applied to calculate polygenic and residual error variance components for heritability (Wang et al., 2016) as follows: *y* = *X*α + φ + ε, where *y* = phenotypic vector, *X* = incident matrix for fixed effects, α = vector of fixed effects, φ∼MVN(0,Kσ*_*g*_*^2^) = polygenic effect with a multivariate normal distribution with zero mean, and ε∼MVN(0,Iσ*_*e*_*^2^) = vector of residues. Moreover, σ*_*g*_*^2^, σ*_*e*_*^2^, and K were used as polygenic variance, residual variance, and kinship matrix, respectively. The above two variance components were estimated from the restricted maximum likelihood (REML) method, whereas the kinship matrix was calculated from marker information (Xu, 2013). Broad-sense heritability was calculated as: $h_{B}^{2}=\frac{\sigma_{g}^{2}}{\sigma_{g}^{2}+\sigma_{e}^{2}}$

### DNA Extraction and Quantification

Total genomic DNA of the 94 tobacco accessions was isolated from 0.1 g fresh young leaves. DNA extraction was performed using a NuClean Plant Genomic DNA Kit (CWBIO, Beijing, China), according to the manufacturer’s protocol. DNA quality and concentration were evaluated using 1% agarose gel electrophoresis and a NanoDrop spectrophotometer. The DNA concentration was normalized to 30 ng/uL for library construction.

### Genotyping-by-Sequencing Library Construction, Sequencing, and Single-Nucleotide Polymorphism Calling

For GBS library preparation, genomic DNA was digested with the restriction enzyme *ApeK1*, and libraries with 250–500 bp were constructed in 96-plex using the protocols developed by Elshire et al. (2011). The GBS libraries were sequenced on an Illumina HiSeq^TM^ 2000 instrument. Raw reads were de-multiplexed using a barcode sequence, and the adapter sequences were trimmed using the standard Illumina GA Pipeline v1.5. High-quality clean short reads were aligned to the tobacco reference genome, *N. tabacum* Nitab4.5 (Edwards et al., 2017), using Burrows-Wheeler Aligner (BWA, V0.7.12) software (Li and Durbin, 2009). The SNP variants were extracted using the Unified Genotyper module of GATK software (v3.4-46) in multiple samples (McKenna et al., 2010). The extracted variants were filtered using the following filter parameters: -Window 4, -filter “QD < 4.0 | | FS > 60.0 | | MQ < 40.0,” -G_filter “GQ < 20.” ANNOVAR software was used to annotate all filtered high-density SNPs (Wang et al., 2010). The GAPIT software package was used to create the kinship matrix between accessions, kinship matrix heatmap, and physical map of SNPs (Lipka et al., 2012).

### Linkage Disequilibrium and Population Structure Analysis

PLINK v1.90 software (Purcell et al., 2007) was used to analyze the LD by calculating the squared correlation coefficients (R^2^) of SNPs, using minor allele frequency (MAF) ≥0.05 and a missing rate <20%, and the LD plot was generated using R script. The LD decay rate was observed when the average R^2^ decreased to half of its maximum value. The population structure for the 94 accessions was evaluated using STRUCTURE v2.3.4 (Pritchard et al., 2000). The hypothetical subgroup (K) values were set from 2 to 10, with 20,000 iterations for each run, followed by 200,000 Markov chain Monte Carlo (MCMC) replications after burn-in. According to Evanno et al. (2005), the best K was identified using STRUCTURE HARVESTER (Earl and vonHoldt, 2012). A neighbor-joining phylogenetic tree of the 94 accessions was constructed using the filtered SNPs by the Tassel 5.2 software (Bradbury et al., 2007).

### Genome-Wide Association Mapping

Genome-wide association studies used SNPs with less than 20% missing data, MAF > 0.05, and sequencing depth ≥3. Four multi-locus (ML) GWAS approaches were used to identify significant QTNs, including mrMLM (Wang et al., 2016), pLARmEB (Zhang et al., 2017), ISIS EM-BLASSO (Tamba et al., 2017), and FASTmrMLM (Tamba and Zhang, 2018), while the Q and K matrix were incorporated into a MLM. These methods were implemented using the R package mrMLM (version 4.0.2).^2^ All multi-locus genome-wide association study (ML-GWAS) models use a modified Bonferroni; the number of markers is replaced by the effective number of markers in the correction formulas (Wang et al., 2016; Zhang et al., 2017). Two-step algorithms are involved in all these methods. In the first step, the single (SL) GWAS method scans the entire genome, with putative QTNs identified according to a less stringent threshold level. In the second step, the effects of selected markers are estimated by empirical Bayesian, the significance of the effects from zero were obtained using the likelihood ratio test, and the threshold level LOD ≥ 3 (*P* = 0.0002) was used to determine significant trait-associated QTNs (Wang et al., 2016; Tamba et al., 2017).

### Superior Allele Analysis for Tobacco Bacterial Wilt Resistance

For this purpose, we used stable QTNs identified in multiple environments and/or by multiple GWAS methods. The resistance allele of each stable QTN was determined using code 1 for genotype and QTN effect value. If the QTN effect value is negative, the genotype with code 1 is considered the TBW-resistant superior allele; if the QTN effect value is positive, the alternative genotype is considered the TBW-resistant superior allele (Wang et al., 2016; Zhang et al., 2019). Correlation coefficients between TBW-DI and the number of superior alleles were calculated using R4.0.3 (see text footnote 1) software. The TBW superior allele percentage was calculated for each accession as the number of superior alleles divided by the total number of stable QTNs. For each QTN, the TBW superior allele percentage in the GWAS population was calculated as the number of accessions with superior alleles divided by the total number of accessions. The best parental cross combinations for tobacco breeding programs were predicted using TBW-resistant superior alleles and stable QTN information.

### Prediction of Potential Candidate Genes

The search for potential candidate genes based on the stable QTNs detected by multiple methods and/or in multiple environments/BLUP was performed using the *N. tabacum* Nitab4.5 reference genome^3^, according to the genome-wide LD decay distance (Edwards et al., 2017). Next, homologous genes related to bacterial wilt in *Arabidopsis* were determined by BLAST analysis with 1E-30 critical *E*-value. These candidate genes were assigned to different biological processes related to bacterial wilt based on the function of their homologs in *Arabidopsis* in literature, such as WRKY transcription factors (TFs) (Cai et al., 2015; Hussain et al., 2018), ethylene-responsive factors (Gutterson and Reuber, 2004), pathogenesis-related proteins (PRs) (Kuhn et al., 2017), Cytochrome P450 family (Li et al., 2021), and brassinosteroids (Sun and Li, 2017). KEGG analysis was used to identify the functional categories (metabolic or signal transduction pathways) of predicted candidate genes, using the KOBAS v3.0 tool^4^, with *P*-value < 5% as threshold criteria (Xie et al., 2011).

## Results

### Phenotypic Variation of Tobacco Bacterial Wilt in a Natural Population

The mean values for TBW-DI across 94 accessions in the four environments (E1–E4) were 52.75, 51.23, 13.34, and 72.04, with SDs of 35.24, 29.42, 16.88, and 26.47, respectively (Table 1). The CVs were >50% in all environments except E4 (36.74%), indicating the highly dispersed distribution of TBW-DI among accessions. The frequency distribution for TBW-DI in the four environments and BLUP is in Supplementary Figure 1. Skewness and kurtosis values were <1 in E1, E2, and E4, indicating that TBW-DI followed a normal distribution; in E3, TBW-DI was skewed slightly to the left (Table 1 and Supplementary Figure 1). The two-way ANOVA exhibited significant differences (*P* < 0.001) for genotype, environment, and genotype × environment interaction, suggesting that environmental factors also influence TBW-DI (Table 1). Moreover, the heritability estimates for TBW-DI in the four environments ranged from 61.37 to 81.36%, using residual and polygenic variances (Table 1), indicating that genetic effects play a significant role in TBW-DI variation.

**TABLE 1:** Statistical analysis of TBW-DI in 94 tobacco accessions in four environments.

Env.	Mean	Range	SD	CV (%)	Skew	Kur	F_*G*_	F_*E*_	F_*G*__×__*E*_	h^2^B (%)
**E1**	52.75	0.00–100	35.24	66.80	−0.05	−1.38	3.42^∗∗^	117.76^∗∗^	14.86^∗∗^	71.07
**E2**	51.23	0.00–100	29.42	57.39	−0.1	−1.22				77.78
**E3**	13.34	1.39–79.44	16.88	78.75	1.53	1.54				81.36
**E4**	72.04	2.78–100	26.47	36.74	−0.76	−0.32				61.37

Env: environments, E1: Nanxiong (2013), E2: Nanxiong (2014), E3: Xikou (2014), E4: Nanxiong (2015), SD: standard deviation, CV: coefficient of variation, Skew: skewness, Kur: kurtosis, F_*G*_, F_*E*_, and F_*G*__×__*E*_: *F*-values for genotype, environment, and genotype × environment, respectively, h^2^B: broad sense heritability. **Significance at the 0.01 level.

### Genotyping-by-Sequencing of the Test Population

We obtained 1412.73 million raw Illumina sequencing reads for the 94 accessions from the GBS library. After quality control and data filtering, 1370.27 million clean reads were generated, with an average 97.06% effective rate (Supplementary Table 2). On average, 98.63% of clean reads had a base error rate of <1% (Q20), and 95.90% of the reads had a base error rate of <0.1% (Q30), with an average GC distribution of 40.92%. Overall, 4.81–23.54 million high-quality sequencing reads were obtained per sample from clean reads, with an average of 9.76 million reads (Supplementary Table 2). Finally, 4.45–22.06 million reads were aligned to the reference genome, with an average mapping ratio of 93.01% (Supplementary Table 2).

**FIGURE 2:**
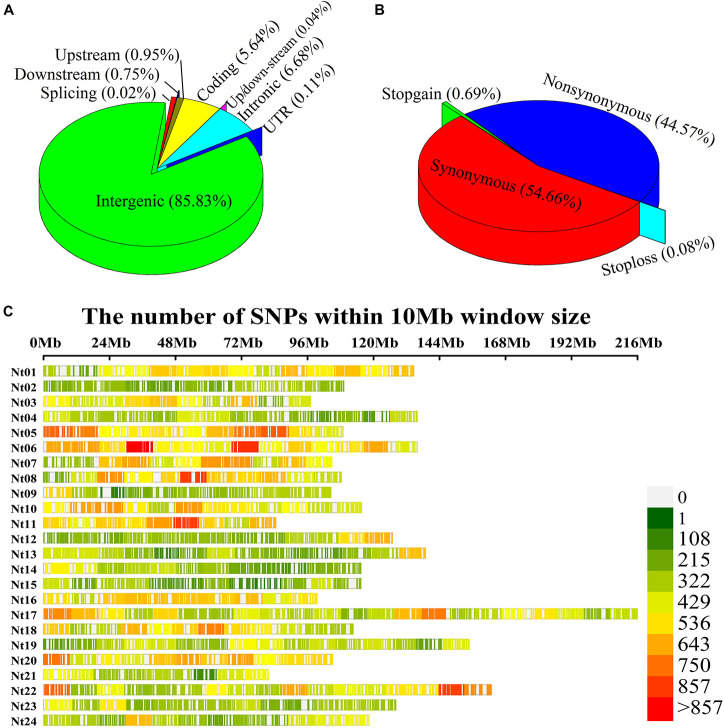
Single-nucleotide polymorphism (SNP) annotation, density, and distribution on 24 chromosomes of tobacco. **(A)** SNP variant classification; **(B)** Coding variant classification; **(C)** Tobacco physical map developed using GBS-SNPs. *Y*-axis represents chromosomes from Nt01 to Nt24, and *X*-axis represents the physical positions of the SNPs on each chromosome.

After completing the sequencing, 938,799 SNP variants were called from GBS sequencing data using the GATK process. Among these, 573,312 SNPs were transitions, and 365,487 SNPs were transversions. The SNP data were filtered with MAF ≥ 5%, missing rate <20%, sequence depth ≥ 3 to obtain 126,602 high-quality SNPs, comprising 90,276 transitional and 36,326 transversional SNPs. SNP functional annotation revealed that most identified SNPs were located within intergenic regions (85.83%) of the genome followed by intronic regions (6.68%), coding variants (5.64%), upstream (0.95%), downstream (0.75%), and UTR regions (0.11%) (Figure 2A). Further classification of coding SNPs revealed that 54.66 and 44.56% are synonymous and non-synonymous, while stop-gain and stop-loss constituted <1% (Figure 2B). Moreover, the SNPs mentioned earlier were distributed on all 24 chromosomes of tobacco (Figure 2C), with an average of 5275.08 SNPs per chromosome (Supplementary Table 3). The maximal number of SNPs were identified on chromosome Nt17 (9583), while those with minimal numbers were on chromosome Nt02 (2966). The average marker density was approximately 24.46 kb/SNP at the genome level (Supplementary Table 3). The highest marker density (16.23 kb/SNP) was on chromosome Nt11, while the lowest marker density (37.94 kb/SNP) was on chromosome Nt15 (Supplementary Table 3). These results demonstrate the uneven distribution of markers throughout the tobacco genome.

### Population Structure, Linkage Disequilibrium, and Kinship Analysis

**FIGURE 3:**
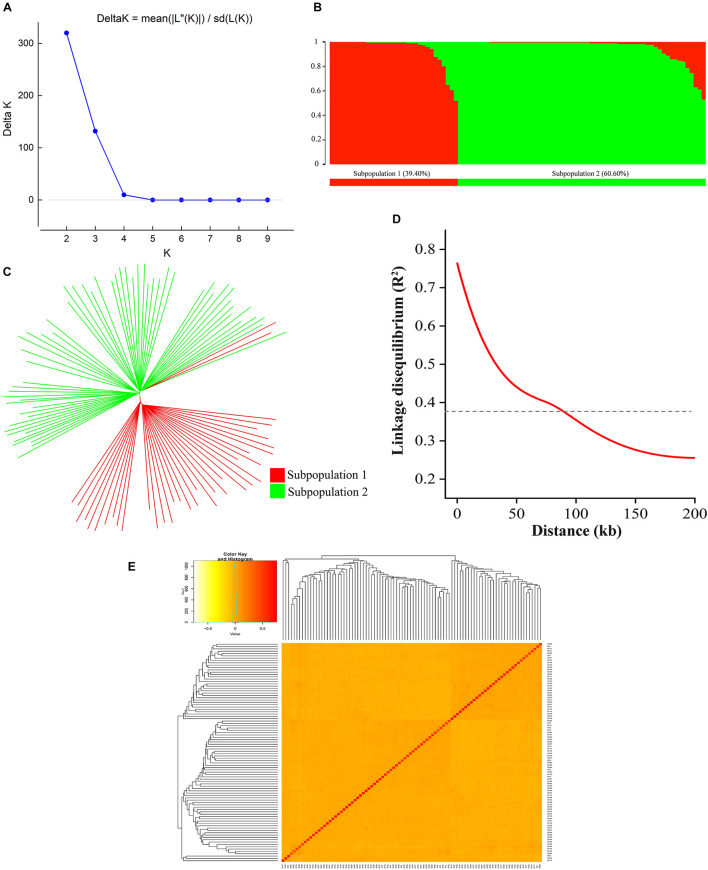
Population structure, phylogenetic tree, LD decay, and kinship of 94 tobacco accessions. **(A)** Relationship between K and Delta K, and determination of subpopulations using Delta method of Evanno et al. (2005); **(B)** Distribution of accessions into subgroups: red and green bars indicate subgroup I and subgroup II, respectively; **(C)** Neighbor-joining phylogenetic tree based on Nei’s genetic distances; **(D)** Entire genome LD decay of the population; **(E)** Heatmap of kinship matrix of 94 accessions.

A total of 126,602 high-density SNPs were used to define the subgroups/subpopulations within the panel of 94 tobacco accessions. Delta K (*K* = 1–10) analysis revealed two subpopulations (selected *K* = 2) comprising 37 (39.40%) and 57 (60.60%) tobacco accessions, respectively (Figures 3A,B). Each subpopulation comprised accessions from different ecological zones (Figure 3B), indicating that the division of two subpopulations was not related to their geographical origins. Furthermore, a neighbor-joining phylogenetic tree was conducted based on their genetic distances derived from the SNP differences in these accessions. The population could be divided into two subpopulations (Figure 3C), and the phylogenetic analysis agreed well with the clustering results in STRUCTURE. The squared correlation coefficient (*r*^2^) values were calculated for all SNP pairs to determine LD decay. The *r*^2^-values decreased rapidly with increasing physical distance between pairwise SNPs (Figure 3D). The overall LD decay distance for all chromosomes was estimated at ∼94.56 kb, where *r*^2^ = 0.381 decreased to half its maximum value (Figure 3D). Moreover, the pairwise relative kinship coefficients showed a lower level of genetic relatedness among 94 tobacco accessions (Figure 3E).

### Identification of Quantitative Trait Nucleotides by Multi-Locus Genome-Wide Association Study Methods

**FIGURE 4:**
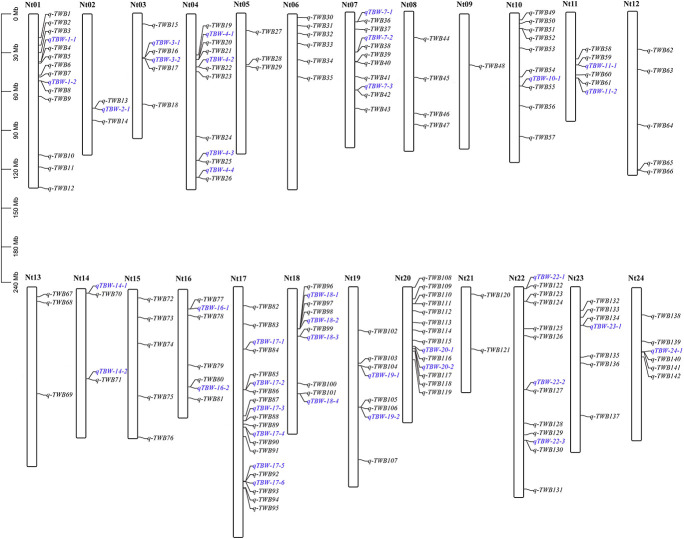
Genetic map based on identified QTNs. The black font represents QTNs identified in E1–E4 and BLUP using four ML-GWAS approaches. The blue font represents QTNs repeatedly identified across at least two environments and/or two ML-GWAS methods.

The four multi-locus methods identified 142 significant QTNs associated with TBW resistance based on LOD scores ≥3 in the four environments and BLUP (Figure 4 and Supplementary Figure 2). Of these, 26, 34, 38, 26, and 28 QTNs were detected in E1, E2, E3, E4, and BLUP, respectively, explaining 8.18, 7.95, 7.03, 7.93, and 8.22% of the phenotypic variation (PVE) on average (range 0.49–22.52%) (Table 2). The corresponding LOD scores ranged from 3.20 to 12.41, 3.02 to 14.19, 3.01 to 15.20, 3.02 to 13.23, and 3.11 to 13.26 (Supplementary Figure 2). Among the 142 QTNs, 7–12, 5–13, 8–17, and 10–13 QTNs were identified using FASTmrMLM, ISIS EM-BLASSO, mrMLM, and pLARmEB, respectively, in E1–E4 and BLUP (Table 2). The corresponding PVE values ranged from 1.56 to 22.52, 1.14 to 19.16, 0.63 to 20.32, and 0.49 to 19.26, and LOD values ranged from 3.02 to 12.11, 3.10 to 9.56, 3.02 to 14.18, and 3.01 to 15.20 (Table 2). Significant QTNs were disseminated on 24 chromosomes, with more than eight QTNs located on chromosomes 1, 4, 7, 10, 17, 20, and 22 (Supplementary Figure 2). Additionally, the QTNs identified in this study were not located in or overlapped with the genomic region of previously reported QTLs for bacterial wilt resistance in tobacco.

**TABLE 2:** Summary of QTNs detected in multiple environments and BLUP model using four ML-GWAS methods.

Method	Total	E1	E2	E3	E4	BLUP	QTN effect	LOD score	*r*^2^ (%)
Total	142	26	34	38	26	28	−37.02 to 30.64	3.01–15.20	0.49–22.52
FASTmrMLM	45	12	8	7	11	9	−17.91 to 20.13	3.02–12.11	1.56–22.52
ISIS EM-BLASSO	44	10	13	10	5	9	−16.01 to 17.76	3.10–9.56	1.14–19.16
mrMLM	54	10	8	17	12	9	−31.02 to 20.12	3.02–14.18	0.63–20.32
pLARmEB	57	10	11	13	12	13	−17.29 to 30.64	3.01–15.20	0.49–19.26

*E1: Nanxiong (2013), E2: Nanxiong (2014), E3: Xikou (2014), E4: Nanxiong (2015), r^2^: phenotypic variance explained by each QTN.*

### Environment- and Method-Stable Quantitative Trait Nucleotides for Tobacco Bacterial Wilt Resistance

**FIGURE 5:**
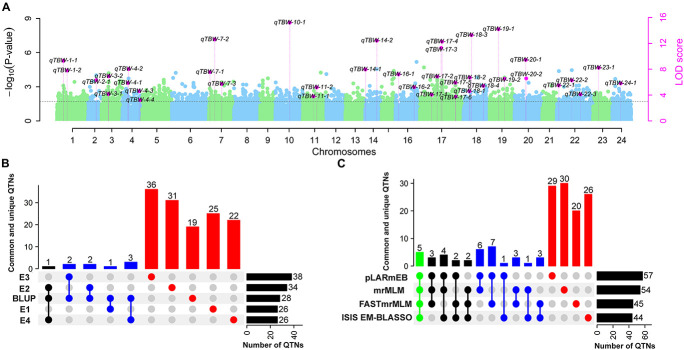
Number of stable QTNs detected for TBW resistance using different ML-GWAS methods in four environments and their BLUP values. **(A)** Pink dots represent stable QTNs identified by more than two methods and/or two environments/BLUP. Light blue and green dots represent the –log10 (*P*-value) of 126,602 markers on 24 chromosomes in the first step of ML-GWAS methods. The threshold level LOD ≥ 3 (*P* = 0.0002) was used to determine significant QTNs and estimated by empirical Bayesian in the second step of ML-GWAS methods; **(B)** Common and unique QTNs identified in E1–E4 and BLUP; **(C)** Common and unique QTNs detected by mrMLM, FastmrMLM, pLARmEB, and ISIS EM-BLASSO. The connected circles below the histogram show overlapping QTNs; green, black, and blue represent four, three, and two overlapping QTNs, respectively, while red represents unique QTNs. Horizontal bars show the total number of QTN set sizes.

Two types of QTNs were defined as stable QTNs: those detected in at least two environments/BLUP (environment-stable) and/or by at least two ML-GWAS models (method-stable). In this study, 38 QTNs were identified as stable for TBW resistance (Table 3 and Figures 4, 5A–C), of which nine were environment-stable (Table 3 in bold and Figure 5B), 37 were method-stable (Table 3 and Figure 5C), and eight were co-detected as environment-stable and method-stable. For example, *qTBW-20-1* was detected in E2, E4, and BLUP by three methods, with LOD and PVE values ranging from 3.10 to 9.43 and 8.05 to 19.98, respectively. Likewise, six stable QTNs, *qTBW-7-1*, *qTBW-7-2*, *qTBW-11-1*, *qTBW-18-3*, *qTBW-18-4*, and *qTBW-19-1* were identified in two environments using two, three, two, four, four, and two ML-GWAS methods; their respective LOD values were 4.55–7.55, 3.49–12.60, 3.37–3.78, 3.86–13.27, 3.10–5.40, and 4.78–14.18, and PVE values were 8.42–17.26, 5.79–16.87, 0.49–1.17, 4.77–14.83, 3.91–12.75, and 4.03–13.37 (Table 3). Three QTNs (*qTBW-2-1*, *qTBW-14-2*, and *qTBW-17-1*) were detected by all four multi-locus methods in one environment, and eight QTNs (*qTBW-3-1*, *qTBW-10-1*, *qTBW-14-1*, *qTBW-17-4*, *qTBW-19-2*, *qTBW-22-3*, *qTBW-23-1*, and *qTBW-24-1*) were detected by three methods in one environment. Interestingly, only one QTN (*qTBW-22-1*) was identified by a single method in two environments, with LOD 4.06–5.48 and PVE 7.03–11.16 (Table 3).

**TABLE 3:** Significant QTNs for TBW resistance detected by multiple ML-GWAS methods and/or in multiple environments/BLUP.

QTN name^*a*^	Chr.	Position (bp)	Effect	LOD score	r^2^ (%)^*b*^	MAF^*c*^	Environment^*d*^	Method^*e*^
*qTBW-1-1*	Nt01	37016222	−16.95 to −16.82	5.62–9.31	12.86–14.41	0.29	E1	M1, M3
***qTBW-1-2***	**Nt01**	**51696366**	**4.87 to 5.91**	**3.54**–**7.79**	**2.50–5.07**	**0.48**	**E3, BLUP**	**M3, M4**
*qTBW-2-1*	Nt02	72820038	13.19 to 15.98	3.56–6.30	9.68–14.06	0.23	E1	M1, M2, M3, M4
*qTBW-3-1*	Nt03	34243911	4.64 to 6.30	3.42–4.16	4.17–6.69	0.44	BLUP	M1, M2, M4
*qTBW-3-2*	Nt03	35123140	11.94 to 15.35	4.58–6.90	6.71–12.29	0.32	E1	M2, M3
*qTBW-4-1*	Nt04	35325384	5.67 to 8.68	3.53–5.87	1.56–5.64	0.49	E4	M2, M3
*qTBW-4-2*	Nt04	40894957	15.10 to 16.36	6.70–8.02	12.60–13.35	0.21	E1	M2, M3
*qTBW-4-3*	Nt04	113176005	−8.05 to −7.19	3.93–4.60	5.06–6.22	0.28	E4	M2, M3
*qTBW-4-4*	Nt04	126224139	−6.35 to −5.23	3.22–5.91	7.05–10.77	0.38	BLUP	M1, M4
***qTBW-7-1***	**Nt07**	**7571216**	**5.21** to **14.57**	**4.55**–**7.55**	**8.42–17.26**	**0.20**	**E2, BLUP**	**M2, M3**
***qTBW-7-2***	**Nt07**	**30847693**	**7.17** to **11.31**	**3.49**–**12.60**	**5.79–16.87**	**0.43**	**E3, BLUP**	**M1, M3, M4**
*qTBW-7-3*	Nt07	60177410	−15.01 to −9.73	3.39–5.72	5.24–12.35	0.21	E1	M2, M4
*qTBW-10-1*	Nt10	56383019	17.11 to 30.64	4.88–15.20	9.21–19.26	0.48	E3	M1, M3, M4,
***qTBW-11-1***	**Nt11**	**40714656**	**3.50** to **10.41**	**3.37**–**3.78**	**0.49–1.17**	**0.45**	**E4, BLUP**	**M1, M3**
*qTBW-11-2*	Nt11	50906143	7.31 to 9.58	4.51–5.18	4.11–7.95	0.28	E4	M1, M2
*qTBW-14-1*	Nt14	3465200	−9.70 to −5.04	3.85–7.95	3.38–17.79	0.14	E3	M2, M3, M4,
*qTBW-14-2*	Nt14	69653438	−14.26 to −8.99	3.98–12.41	3.12–8.68	0.25	E1	M1, M2, M3, M4
*qTBW-16-1*	Nt16	15141918	13.11 to 15.35	5.87–7.20	11.04–19.16	0.14	E4	M2, M4
*qTBW-16-2*	Nt16	75282825	−12.77 to −10.82	3.31–5.21	5.22–9.98	0.42	E4	M1, M3
*qTBW-17-1*	Nt17	48243892	11.26 to 17.76	4.05–12.11	13.97–22.52	0.17	E4	M1, M2, M3, M4
*qTBW-17-2*	Nt17	79641667	10.73 to 11.80	3.25–6.85	5.08–6.78	0.35	E1	M1, M3
*qTBW-17-3*	Nt17	103649413	4.74 to 6.53	6.59–11.28	8.06–13.36	0.43	BLUP	M1, M2
*qTBW-17-4*	Nt17	108558750	−14.37 to −6.04	3.61–12.26	1.93–8.92	0.42	E1	M1, M2, M4,
*qTBW-17-5*	Nt17	172367035	5.42 to 5.66	3.99–6.90	8.31–9.35	0.33	BLUP	M1, M3
*qTBW-17-6*	Nt17	172718671	−10.87 to −10.37	3.59–3.75	3.23–3.26	0.45	E1	M2, M3
*qTBW-18-1*	Nt18	31032756	−10.05 to −7.92	4.59–5.29	5.29–12.68	0.07	E3	M2, M3
*qTBW-18-2*	Nt18	31188535	4.93 to 7.03	5.68–6.69	8.62–15.28	0.22	BLUP	M1, M2
***qTBW-18-3***	**Nt18**	**36818226**	**5.99 to 19.46**	**3.86–13.27**	**4.77–14.83**	**0.47**	**E4, BLUP**	**M1, M2, M3, M4**
***qTBW-18-4***	**Nt18**	**81285311**	−**11.26 to**−**3.64**	**3.10–5.40**	**3.91–12.75**	**0.36**	**E4, BLUP**	**M1, M2, M3, M4**
***qTBW-19-1***	**Nt19**	**60933888**	**3.60 to 14.06**	**4.78–14.1**	**4.03–13.37**	**0.48**	**E2, BLUP**	**M1, M3, M4**
*qTBW-19-2*	Nt19	92990637	−19.86 to −17.29	5.10–6.53	5.80–6.92	0.47	E1	M1, M2, M3,
***qTBW-20-1***	**Nt20**	**45986131**	−**14.90 to**−**5.04**	**3.10–9.43**	**8.05–19.98**	**0.21**	**E2, E4, BLUP**	**M2, M3, M4**
*qTBW-20-2*	Nt20	47568998	3.51 to 5.26	3.78–6.57	1.77–6.66	0.35	E3	M1, M3
***qTBW-22-1***	**Nt22**	**1437500**	**6.46 to 12.37**	**4.06–5.48**	**7.03–11.16**	**0.14**	**E1, BLUP**	**M4**
*qTBW-22-2*	Nt22	79361894	3.88 to 7.45	3.13–6.27	4.24–14.13	0.37	BLUP	M2, M4
*qTBW-22-3*	Nt22	118588843	7.63 to 9.65	3.59–4.10	0.63–1.14	0.33	E4	M1, M3, M4,
*qTBW-23-1*	Nt23	29888578	10.73 to 20.12	3.68–8.22	1.97–5.77	0.45	E2	M1, M2, M3,
*qTBW-24-1*	Nt24	49518556	15.12 to 20.13	4.21–5.81	7.32–12.39	0.48	E3	M1, M2, M3,

*Normal font indicates stable QTNs detected by at least two methods. Bold font indicates stable QTNs identified in at least two environments/BLUP. ^*a*^*qTBW*- -: _*QTN*_TBW-chromosome-number. ^*b*^r^2^ (%): phenotypic variance explained by each QTN. ^*c*^MAF: Minor allele frequency. ^*d*^M1: mrMLM, M2: FASTmrMLM, M3: pLARmEB, M5: ISIS EM-BLASSO. ^*e*^E1: Nanxiong (2013), E2: Nanxiong (2014), E3: Xikou (2014), E4: Nanxiong (2015).*

### Identification of Superior Alleles

**FIGURE 6:**
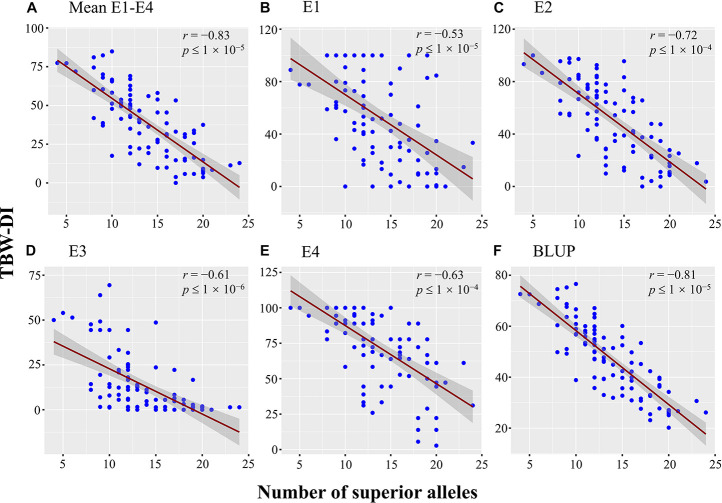
Scatter plot with fitted regression lines and 95% confidence interval bands, showing a negative correlation between TBW-DI in **(A)** Mean E1–E4, **(B)** E1, **(C)** E2, **(D)** E3, **(E)** E4, **(F)** BLUP and number of alleles. The E1, E2, E3, and E4 denote the populations planted in Nanxiong (2013), Nanxiong (2014), Xikou (2014), and Nanxiong (2015), respectively. The *X*-axis represents the number of superior alleles, while the *Y*-axis corresponds to the TBW-DI.

The 38 stable QTNs were used to identify superior alleles for TBW resistance using QTN effect values. Thirty-eight superior alleles were identified and significantly (*P* < 0.05) differed from the alternative alleles (Supplementary Table 4). The TBW-DI values for the accessions with superior alleles ranged from 31.73 to 46.91, while those for the alternative alleles ranged from 47.1 to 72.44 (Supplementary Table 4). For example, *qTBW-14-2* had CC as a superior allele and TT as an alternative allele, and TBW-DI values of 44.22 and 58.04, respectively. Similarly, three stable QTNs, *qTBW-20-2*, *qTBW-22-3*, and *qTBW-23-1*, had TT, CC, and TT superior alleles with TBW-DI values <45 (Supplementary Table 4). Moreover, all TBW-DI values for superior alleles of the 38 stable QTNs were <47; according to the disease index, these alleles are considered TBW-resistant superior alleles. A negative correlation was detected between the number of superior alleles and TBW-DI (*r* = −0.83, *p* ≤ 1 × 10^–5^) (Figure 6A). A similar trend was found between the number of superior alleles and TBW-DI in E1 (−0.53, *p* ≤ 1 × 10^–5^), E2 (*r* = −0.72, *p* ≤ 1 × 10^–4^), E3 (*r* = −0.61, *p* ≤ 1 × 10^–6^), E4 (*r* = −0.63, *p* ≤ 1 × 10^–4^), and BLUP (*r* = −0.81, *p* ≤ 1 × 10^–5^) (Figures 6B–F). Based on these results, the superior alleles can be used in MAS for TBW resistance in tobacco.

### Distribution of Superior Alleles and Prediction of Best Cross Combination for Tobacco Bacterial Wilt Resistance

The number of TBW-resistant superior alleles for each stable QTN in the 94 accessions ranged from 1 (1.06%) to 91 (96.81%). Among the 38 stable QTNs, 11 had >50% superior alleles, and 27 had <50% superior alleles; only four had >80% superior alleles (Supplementary Table 4). The number of superior alleles for each accession ranged from 4 (10.52%) to 24 (63.15%); 15 accessions had >50% superior alleles, and 79 accessions had <50% superior alleles. In addition, K326, Ruyuan No. 1, MC133, C176, ROX28, CO258, H66B, and RG17 had 24, 21, 20, 20, 20, 20, 18, and 18 TBW-resistant superior alleles. These accessions can be used in tobacco breeding programs to increase the number of superior alleles for TBW resistance in one cultivar. For example, a cross between K326 (24 TBW-resistant superior alleles) and C176 (20 TBW-resistant superior alleles) could produce offspring with 30 superior alleles. Based on this information, we predicted the following best parental cross combinations: K326 × C176, MC133 × H66B, MC133 × Ruyuan No. 1, CO258 × ROX28, and CO258 × RG17.

### Candidate Genes Underlying Stable Quantitative Trait Nucleotides for Tobacco Bacterial Wilt Resistance

The genomic regions (±95 kb around the associated QTNs) of QTN-linked candidate genes were adopted according to the genome-wide LD decay distance (about 94.5 kb) in this study. As a result, 642 genes were presented in the above regions of the 38 stable QTNs, according to *N. tabacum* reference genome Nitab4.5, of which 489 were homologous in *Arabidopsis*. These 489 genes belonged to different functional categories: stress and defense-related, unknown functional families, hormonal signaling, transcription, translation, transporter, and cell metabolism. Further, the Kyoto encyclopedia of genes and genomes (KEGG, see text footnote 4) analysis of the above 642 genes indicated that 74 genes were involved in 19 KEGG pathways (adjusted *P*-value ≤ 0.05), including flavonoid biosynthesis, glutathione metabolism, MAPK signaling pathway, phenylpropanoid biosynthesis, phosphatidylinositol signaling system, gingerol biosynthesis, and plant–pathogen interactions. Finally, 52 genes were considered potential candidate genes associated with disease resistance in plants (Supplementary Table 5) based on functional annotation, homologous to known genes, and pathway enrichment analysis. For example, candidate gene *Nitab4.5_0002694g0030* underlying the stable QTN *qTBW-3-2* was homologous to *AT4G34050* (*CCoAOMT1*), which annotates caffeoyl-CoA O-methyltransferase and phenylpropanoid in *Arabidopsis*. Similarly, *Nitab4.5_0000274g0070* and *Nitab4.5_0000123g0350*, located near *qTBW-17-3* and *qTBW-24-1*, respectively, corresponded with *Arabidopsis thaliana* genes *AT5G15130* (*WRKY72*) and *AT2G40890* (*CYP98A3*) involved in diarylheptanoid, stilbenoid, gingerol biosynthesis, phenylpropanoid biosynthesis, and flavonoid biosynthesis (Supplementary Table 5). Therefore, these candidate genes may regulate tobacco growth to increase plant defense and disease resistance.

## Discussion

To study the mechanism of TBW resistance in tobacco plants, GWAS is a useful tool for dissecting the genetic basis and candidate genes for the natural variations in a targeted quantitative trait (Zhang et al., 2019). Here, four ML-GWAS methods were used to analyze TBW-DI and BLUP values using 126,602 high-density SNP markers. We identified 38 stable QTNs, superior alleles, and 52 candidate genes associated with TBW resistance. The markers associated with TBW resistance can be used to develop resistant varieties.

### Population Selection for Association Mapping

To gain some insight, we evaluated 94 tobacco accessions for TBW in four different environments at two locations; the variance components indicated that TBW is affected by environmental conditions. The broad-sense heritability for TBW-DI was moderate (61–81%) and differed between environments (Table 1). These observations are similar to other studies in tobacco (Nishi et al., 2003; Qian et al., 2013; Lan et al., 2014), including some heritability problems (Gao et al., 2010).

Genetic diversity in modern tobacco cultivars is low (Wernsman, 1999; Leng et al., 2010); only a few genotypes are ancestors of most cultivars. Thus, it is challenging to assemble a natural population with rich genetic diversity. However, based on the 126,602 SNPs, we found 24.46 kb/SNP in the whole genome (Supplementary Table 3 and Figure 2C) and higher coverage density than reported elsewhere (Leng et al., 2010; Wang et al., 2021). We also found an LD decay distance of ∼94.56 kb (Figure 3D), much smaller than that reported by Fricano et al. (2012). STRUCTURE analysis identified two subpopulations in the panel (Figure 3B), and clustering results showed that the genetic background of 94 accessions is diverse and complex, consistent with most studies on tobacco (Fricano et al., 2012; Dadras et al., 2014; Wang et al., 2021). There are high genome-wide SNP variations in the panel used in this study that are suitable for association mapping (Mackay and Powell, 2007; Wang et al., 2009; Ikram et al., 2020).

### Statistical Power of Multi-Locus Genome-Wide Association Methods and Significance of Stable Quantitative Trait Nucleotides

The four ML-GWAS methods—FASTmrMLM, ISIS EM-BLASSO, mrMLM, and pLARmEB—identified 45, 44, 54, and 57 significant QTNs for TBW-DI, with small to large effects (Table 2). While the statistical power of QTN detection has improved, after controlling the polygenic background, most small-effect QTNs of complex traits are not captured by SL-GWAS methods (Zhang et al., 2019). However, ML-GWAS studies have shown that these methods have high-resolution power; e.g., Hou et al. (2018) used SL- and ML-GWAS models to identify 20 QTNs related to the drought stress response using mrMLM, but only three by EMMAX, suggesting that the ML-GWAS methods are more powerful. Likewise, Su et al. (2018) reported that multi-locus methods are robust and more potent than MLM method.

In this study, 38 QTNs were identified in more than two environments and/or ML-GWAS models (Table 3 and Figures 4, 5); nine were considered environment-stable, 37 were considered method-stable, and eight were considered both. In previous studies, environmental-stable QTNs have gained more attention than method-stable QTNs (Zhang et al., 2019), but recent studies have shown that QTNs detected using multiple methods are also reliable (Cui et al., 2018; Li et al., 2018; Peng et al., 2018; Chaurasia et al., 2020; Ikram et al., 2020). For example, 42 QTNs related to salt stress in wheat were detected using multiple methods (Chaurasia et al., 2020). Hence, environment-stable and method-stable QTNs are more reliable for breeding programs, with similar results reported in other crop plants (Zhang et al., 2019), including soybean (Ikram et al., 2020), maize (Ma et al., 2018), wheat (Chaurasia et al., 2020; Danakumara et al., 2021), and rice (Verma et al., 2021). In addition, the 38 stable QTNs identified in our study are considered novel as they are not located in the genomic region of previously reported QTLs for TBW.

### Application of Superior Alleles in Breeding Programs

Tobacco cultivars exhibit low genetic diversity, and their existing alleles may not improve TBW resistance (Qian et al., 2013; Wang et al., 2021). New alleles identified through germplasm screening will improve TBW resistance. Marker-assisted breeding has dramatically improved breeding efficiency. The alleles of stable QTNs significantly differed, with 4–24 superior alleles for TBW resistance found in the 94 accessions (Supplementary Table 4). Eight resistant genotypes were identified with superior alleles for TBW resistance that can be used to breed highly resistant varieties. The best cross combinations were identified based on these superior alleles for TBW resistance, similar to previous studies for complex traits in different crops (Wang et al., 2006; Zeng et al., 2017). The concept of molecular design breeding (Peleman and Van Der Voort, 2003; Zeng et al., 2017) was used by Tian and his co-workers to successfully selected the LYP9 rice variety with high yield and quality by transferring several alleles into the new cultivar (Tian et al., 2009; Zeng et al., 2017).

### Candidate Genes for Tobacco Bacterial Wilt Resistance

The identification of candidate genes associated with quantitatively inherited traits is challenging in genetic research. The present study identified 52 candidate genes underlying the 38 stable QTNs, based on homology with *Arabidopsis* and KEGG pathways for plant defense and disease resistance (Supplementary Table 5). The gene *Nitab4.5_0002694g0030* encodes the caffeoyl-CoA O-methyltransferase-like protein that may prevent TBW by regulating the phenylpropanoid pathway and lignin production (Yang et al., 2017). *Nitab4.5_0001039g0060* is homologous to *CYCD3;2* in *Arabidopsis* (Supplementary Table 5), and *CYCD3* genes appear to be positive regulators of plant resistance because mutations in the target gene conferred increased disease susceptibility to plant pathogens (Hamdoun et al., 2016). Similarly, *Nitab4.5_0000337g0220* encodes BR-signaling kinase 1 (*BSK1*), and a bsk1-1 mutation displayed enhanced susceptibility to a range of pathogens, demonstrating that *BSK1* plays an important role in plant immunity (Shi et al., 2013). *BSK1* is a substrate of the brassinosteroid receptor BRI1 and plays a critical role in brassinosteroid signaling to regulate plant immunity (Tang et al., 2008; Sun and Li, 2017). Most of the candidate genes (*Nitab4.5_0000430g0170*, *Nitab4.5_0000016g0210*, *Nitab4.5_0002576g0050*, *Nitab4.5_0000553g0050*, and *Nitab4.5_0002890g0050*) were involved in signaling pathways, and their homologous genes (*MKK9*, *bZIP65*, *AT1G17345*, *EPF2*, and *EMB14*, respectively) in *A. thaliana* play a significant role in plant disease resistance (Feys and Parker, 2000; Eshraghi et al., 2014; Liu and Lam, 2019). *Nitab4.5_0000641g0050*—involved in the glutathione metabolism pathway and glutathione—is the most abundant antioxidant in cells and crucial for life processes. It protects DNA, biomolecules, and proteins against oxidative damage, which favored resistance against environmental stresses (Freeman et al., 2004; Li et al., 2021) and increased resistance in eggplant after infection with *R. solanacearum* (Avinash et al., 2017). TFs are essential regulatory genes in plants, and WRKY TFs play a significant role in the immune response of plants to various biological stresses (Chen et al., 2017). Two WRKY TF genes (*Nitab4.5_0000929g0030* and *Nitab4.5_0000274g0070*) were identified in this study (Supplementary Table 5). Numerous research findings have shown that *WRKY22* and *WRKY40* TF genes have positive regulatory effects on the resistance of Solanaceae crops to bacterial wilt (Dang et al., 2014; Cai et al., 2015; Hussain et al., 2018). These results suggest that WRKY genes could be important positive regulators of tobacco plant resistance against bacterial wilt (Hussain et al., 2018). The relationship between these candidate genes and TBW resistance needs to be verified.

The fundamental task is to find excellent genes or QTNs related to the target trait to achieve the precise breeding, design, and breed aggregate of excellent genes/alleles. Most researchers have only used linkage analysis or association analysis to identify QTLs or SNPs/QTNs. Previous studies only contain basic theoretical results (Nishi et al., 2003; Lan et al., 2014), with few researchers using these results to screen material. However, pleiotropic genes regulate quantitative traits, their genetic laws are complex (Yang et al., 2012), and it is difficult for a single QTL to reflect the advantages of traits. Here, 38 stable QTNs and 52 candidate genes were detected by GWAS, filling a gap and laying a theoretical foundation for subsequent design and breeding. Using research results to evaluate material phenotypes will assist in selecting material containing multiple superior alleles to increase the probability of selecting material with desired traits, which has important implications for molecular marker-assisted screening. Therefore, this study screened tobacco varieties that carry the target QTN alleles and candidate genes that could be used as resistant parents for gene pyramiding to improve TBW resistance.

## Conclusion

In this study, we used GBS technology for the first time to conduct GWAS for TBW resistance to identify QTNs, superior alleles, and candidate genes for breeding highly resistant tobacco varieties. We demonstrated that TBW resistance is genetically complex. We identified 38 novel stable QTNs with significantly different alleles in the association panel. We predicted the five best parental cross combinations based on superior allele information for developing tobacco varieties that are highly resistant to *R. solanacearum*. Moreover, 52 candidate genes were associated with TBW resistance. The results from this study serve as the basis for resistance gene cloning and further understanding of the molecular mechanisms of tobacco resistance to *R. solanacearum*.

## Data Availability Statement

The sequence read data from the genotyping-by-sequencing (GBS) for the 94 sequenced tobacco accession are available in NCBI Sequence Read Archive (SRA) under the accession number PRJNA759331 (https://www.ncbi.nlm.nih.gov/sra/PRJNA759331).

## Author Contributions

PG conceived and designed the experiments. RLa, RLi, YX, MI, and WZ performed the experiments and analyzed data. YX, MI, RLi, QY, ZZ, and WZ contributed to reagents, materials, and analysis tools. RLa, MI, RLi, YX, KS, and PG wrote the manuscript. All authors have read and agreed to the published version of the manuscript.

## Conflict of Interest

WZ is employed by Nanxiong Research Institute of Guangdong Tobacco Co., Ltd., Nanxiong, China. The remaining authors declare that the research was conducted in the absence of any commercial or financial relationships that could be construed as a potential conflict of interest.

## Publisher’s Note

All claims expressed in this article are solely those of the authors and do not necessarily represent those of their affiliated organizations, or those of the publisher, the editors and the reviewers. Any product that may be evaluated in this article, or claim that may be made by its manufacturer, is not guaranteed or endorsed by the publisher.

## Footnotes

1
http://www.R-project.org/

2
https://cran.r-project.org/web/packages/mrMLM/index.html

3
https://solgenomics.net/organism/Nicotiana_tabacum/genome

4
http://kobas.cbi.pku.edu.cn/kobas3
