# Supplementary material for: Identification of Novel Quantitative Trait Nucleotides and Candidate Genes for Bacterial Wilt Resistance in Tobacco (Nicotiana tabacum L.) Using Genotyping-by-Sequencing and Multi-Locus Genome-Wide Association Studies
Source: Front Plant Sci. 2021 Oct 21;12:744175. doi: 10.3389/fpls.2021.744175 (PMC8566715; doi:10.3389/fpls.2021.744175)
Supplement: Supplementary file 6 [file Image_1.pdf]

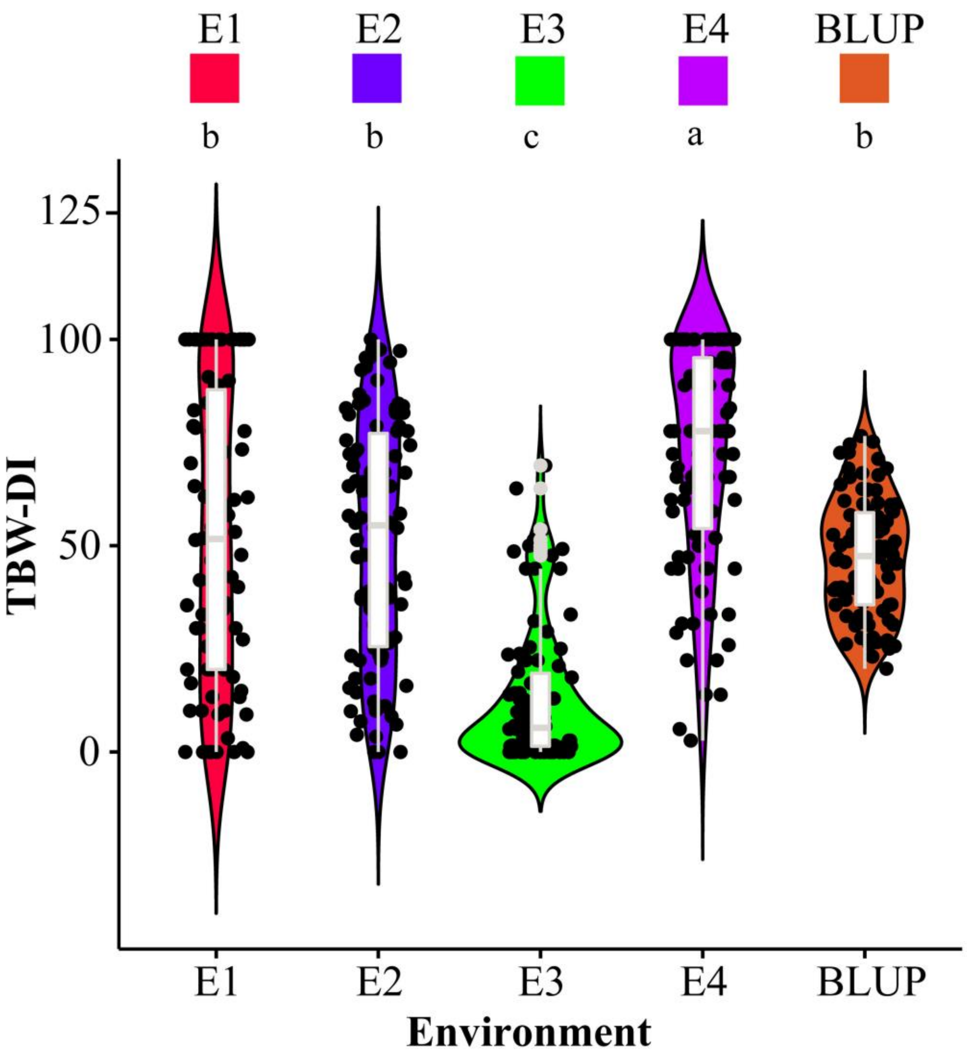

Supplementary Figure 1. Frequency distribution of TBW-DI in 94 accessions in four environments (E1–E4) and BLUP. The significant differences of TBW-DI among four environments/BLUP are tested using the LSD method at the 0.05 level of significance.
