# Supplementary material for: Identification of Novel Quantitative Trait Nucleotides and Candidate Genes for Bacterial Wilt Resistance in Tobacco (Nicotiana tabacum L.) Using Genotyping-by-Sequencing and Multi-Locus Genome-Wide Association Studies
Source: Front Plant Sci. 2021 Oct 21;12:744175. doi: 10.3389/fpls.2021.744175 (PMC8566715; doi:10.3389/fpls.2021.744175)
Supplement: Supplementary file 7 [file Image_2.pdf]

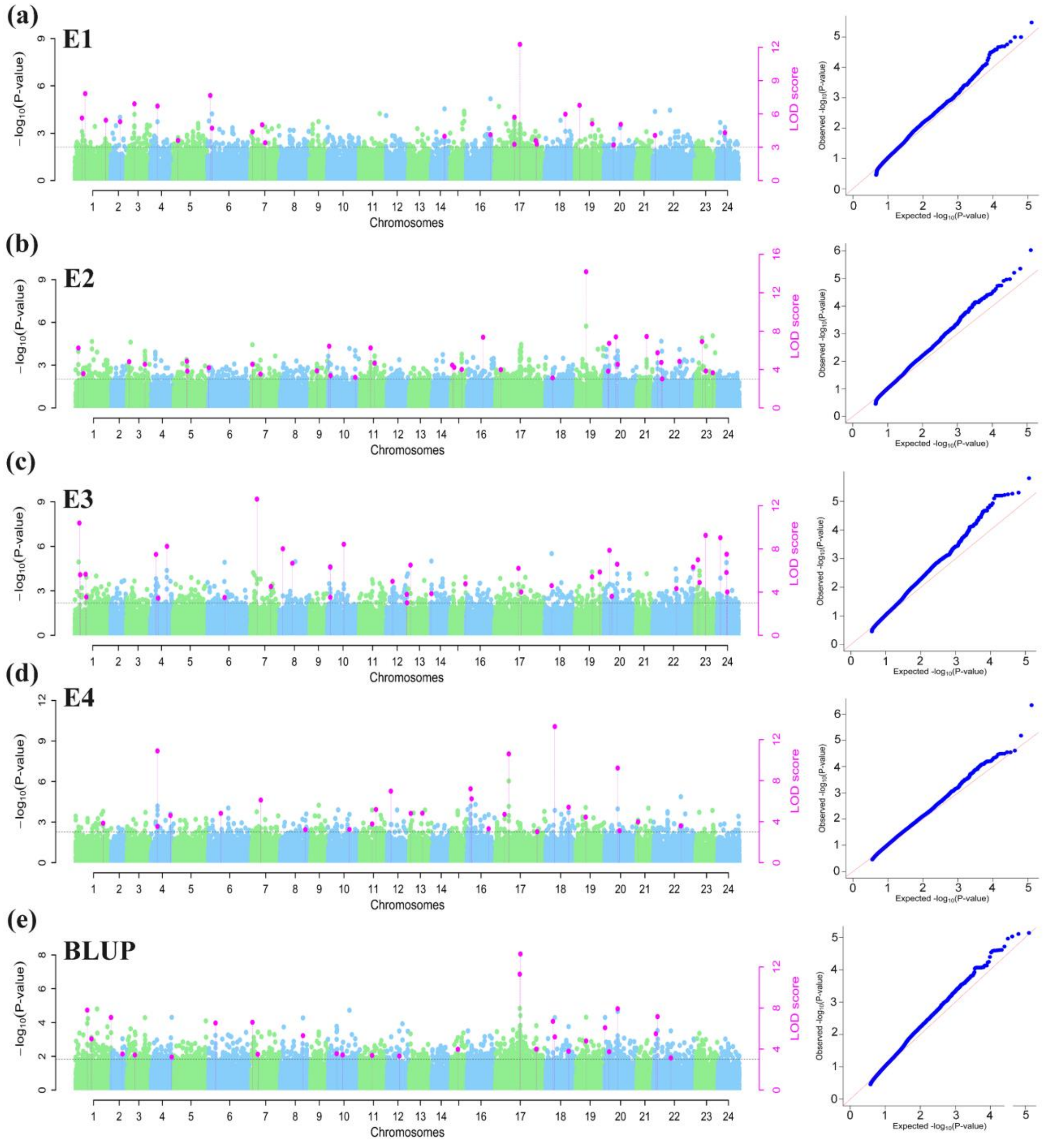

Supplementary Figure 2. Manhattan and q-q plot of association mapping of tobacco bacterial wilt. The small figures (**a–e**) show significant QTNs in four environments and their BLUP values. The pink dots denote significant QTNs in the second step of the multi-locus models, while light green and light blue colors indicate the $–\log_{10}$ *p*-values in the first step. The dashed line on each small figure indicates the LOD score threshold. **E1**: Nanxiong (2013); **E2**: Nanxiong (2014); **E3**: Xikou (2014); **E4**: Nanxiong (2015).
